# Transcriptional Signatures of a Dynamic Epilepsy Process Reveal Potential Immune Regulation

**DOI:** 10.1007/s12035-023-03786-x

**Published:** 2023-11-22

**Authors:** Yanruo Huang, Qihang Wang, Xiaoyin Liu, Wenjie Du, Zijian Hao, Yingwei Wang

**Affiliations:** 1 Department of Anesthesiology, Huashan Hospital, Fudan University, 12 Middle Wulumuqi Road, Shanghai, 200040 People’s Republic of China; 2 State Key Laboratory of Molecular Biology, Shanghai Institute of Biochemistry and Cell Biology, Center for Excellence in Molecular Cell Science, Chinese Academy of Sciences, Shanghai, 200031 People’s Republic of China; 3 University of Chinese Academy of Sciences, Beijing, 100049 People’s Republic of China; 4 Department of Neurosurgery, West China Medical School, West China Hospital, Sichuan University, Chengdu, Sichuan 610041 People’s Republic of China; 5 Institute of Science and Technology for Brain-Inspired Intelligence, Fudan University, Shanghai, 200433 People’s Republic of China; 6 MOE Frontiers Center for Brain Science, Fudan University, Shanghai, 200433 People’s Republic of China

**Keywords:** Epilepsy, Immune cell types, Immune regulation, Molecular mechanism, Transcriptional signatures

## Abstract

Epilepsy is a progression of development and advancement over time. However, the molecular features of epilepsy were poorly studied from a dynamic developmental perspective. We intend to investigate the key mechanisms in the process of epilepsy by exploring the roles of stage-specifically expressed genes. By using time-course transcriptomic data of epileptic samples, we first analyzed the molecular features of epilepsy in different stages and divided it into progression and remission stages based on their transcriptomic features. 34 stage-specifically expressed genes were then identified by the Tau index and verified in other epileptic datasets. These genes were then enriched for immune-related biological functions. Furthermore, we found that the level of immune infiltration and mechanisms at different stages were different, which may result from different types of immune cells playing leading roles in distinct stages. Our findings indicated an essential role of immune regulation as the potential mechanism of epilepsy development.

**Supplementary Information:**

The online version contains supplementary material available at 10.1007/s12035-023-03786-x.

## Introduction

Epilepsy, characterized by recurrent unprovoked seizures, is one of the most common neurological diseases affecting over 70 million people worldwide [1]. The pathogenesis of epilepsy is complex [2]. The imbalance between excitation and inhibition of the central nervous system (CNS), caused by blocking inhibitory conductances or activating excitatory conductances, is an acknowledged mechanism for epilepsy [3]. Besides electrical activity derangement, other mechanisms including epigenetic [4], metabolic [5, 6], and immunologic disturbances also contributed to epilepsy [7]. Furthermore, those epileptogenic abnormalities are conjectured to occur in a cascade, which is involved in the initiation, progression, and maintenance of the epileptogenic process [8]. Since epilepsy is a progress of development and advancement over time, exploring the molecular signatures corresponding to the different stages of epilepsy might offer more clues for studying the CNS function under epilepsy conditions. The genes specifically expressed at different time points might link to different sets of mechanisms. Thus, identification of those genes can not only measure the presence and severity of epilepsy but also reveal the specific mechanisms of the epileptogenic process.

A biomarker is defined as an objectively measured characteristic of various biological processes. Some epilepsy-relevant biomarkers are currently used as effective tools for diagnosis and prediction [9, 10]. Most epilepsy-relevant biomarkers are only specifically expressed in the early periods of epileptogenesis and used as warning signals for epileptic seizures [11]. However, as epilepsy is a gradual development process, molecular signatures during progression and remission may contain more comprehensive information on the pathogenesis of epilepsy. The characteristic genes expressed at the progression stage could indicate an increased risk of ictogenicity, while the genes expressed at the remission stage may imply decreased ictogenicity, even to a “normal” level [1]. Finding the genes involved in the epileptic stage development might help us better understand the mechanisms of this disease.

In this paper, we utilized time-course transcriptomic data of epileptic samples and explore the molecular features during the process of epilepsy with a dynamic developmental perspective. We first distinguished the epileptic state into the progression and remission stages. Then, the Tau index was utilized to define specifically expressed genes among those stages, which enabled the prediction of the progression of epilepsy. Furthermore, we explored the biological functions of those stage-specifically expressed genes and found that they may regulate the process of epilepsy through immune-related pathways. Finally, we demonstrated the roles of these genes in immune infiltration at different stages. These results indicate an essential role of immune regulation as the potential mechanism of epilepsy development, which provides indications for developing novel therapies targeting different stages of epilepsy.

## Materials and Methods

### Data Acquisition and Processing

**Fig. 1:**
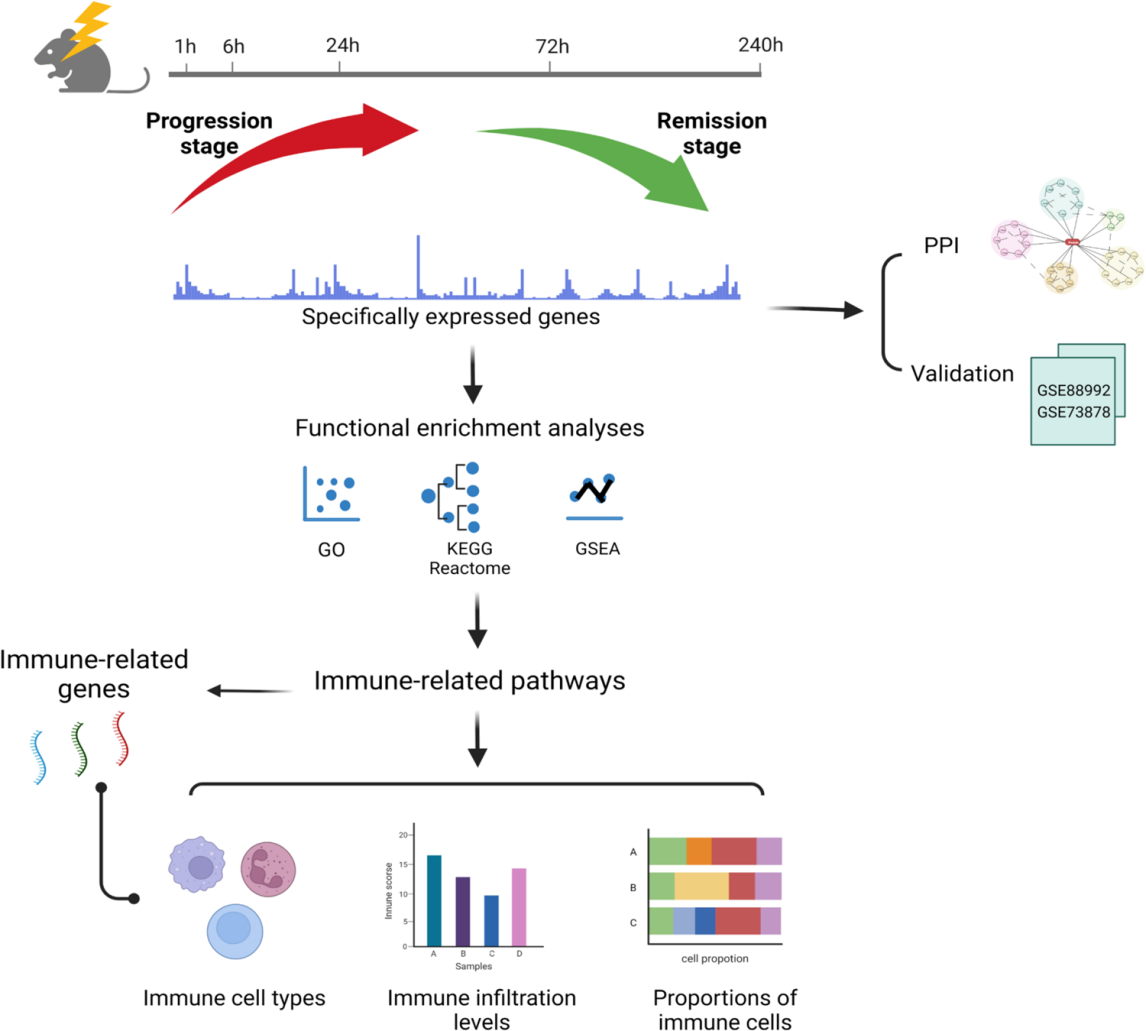
Flowchart for bioinformatics analysis in this study. We utilized time-course transcriptomic data of epilepsy model. The epileptic states were distinguished into the progression and remission stages based on their transcriptomic features at different time points. Then, the specifically expressed genes among those stages were defined. We built the protein–protein interaction (PPI) network of these genes and further validated their prediction performance for different stages of epilepsy using another two datasets. The functional and pathway enrichment of these stage-specifically expressed genes were assessed by Gene Ontology (GO), Kyoto Encyclopedia of Genes and Genomes (KEGG), Reactome, and Gene Set Enrichment Analysis (GSEA) analysis. Finally, the immune-related pathways and genes were selected to illustrate the important immune mechanisms in epilepsy development stage

All data used in the study were obtained from Gene Expression Omnibus (GEO; https://www.ncbi.nlm.nih.gov/gds/). Ethics approval and informed consent were not required. Raw gene expression profiling was accessed from the GSE1834 dataset, which includes the kainic acid (KA)–induced epilepsy model groups and control groups at 5 time points (1, 6, 24, 72, and 240 h after seizure induction). The GSE88992 and GSE73878 datasets served as validation datasets, including epileptic samples at 6 h, 12 h, 24 h, 7 days, 28 days, and 40 days. The workflow chart is shown in Fig. 1.

### Analyses of Sample Heterogeneity

To avoid non-uniform data distribution and data noise, we selected the top 500 genes with the highest standard deviation of the expression level across all samples in the GSE1834 dataset for principal component analysis (PCA). As for Euclidean distance analysis and Pearson’s correlation calculation, which is used for the variation of gene expression profiles and correlation profiles between the control and epilepsy groups at different time points, respectively, all genes from all samples in the GSE1834 dataset were utilized.

### Identification of Stage-Specifically Expressed Genes

The Tau index of gene tissue-specificity was applied to identify genes with specific expression in samples at different stages. Tau was calculated as follows:

$${\mathrm{TAU}}_{\mathrm{max}}=\frac{\sum_{\mathrm{i}=1}^{\mathrm{N}}[\left(1-{X}_{i}/{X}_{max}\right)]}{N-1}$$

$${\mathrm{TAU}}_{\mathrm{min}}=\frac{\sum_{\mathrm{i}=1}^{\mathrm{N}}[\left(1-{X}_{min}/{X}_{i}\right)]}{N-1}$$

N is the number of stages examined. ${X}_{max}$ is the highest expression level and ${X}_{min}$ is the lowest expression level detected for a given gene over all stages examined. ${X}_{i}$ is a given gene expression level of sample $i$. Tau values interpolate the entire range between 0 (for general expression genes) and 1 (for strictly specific genes). We followed the threshold (threshold value ≥ 0.8) to define the expression of specific genes at different stages [12, 13]. The gene heatmap was conducted with TBtools [14].

### Protein–Protein Interaction (PPI)

The interactions of stage-specifically expressed genes were analyzed by the online STRING database [15]. The Cytoscape was used to construct the network of genes, and the plug-in CytoHubba was used to identify hub genes of the PPI network [16].

### Functional Enrichment Analysis

Gene Ontology (GO), Reactome pathway, and Kyoto Encyclopedia of Genes and Genomes (KEGG) enrichment analyses were conducted by the online tool Metascape (http://metascape.org) [17]. GO categories comprised biological processes (BP), molecular functions (MF), and cellular components (CC). We defined significantly changed terms as those fulfilling *p*-value < 0.05. The enrichment maps were visualized with TBtools [14].

### Gene Set Enrichment Analysis (GSEA) and ssGSEA

GSEA was used to assess whether the differential gene sets showed statistical significance between two biological states. The “clusterProfiler” package was used to perform GSEA for the potential mechanism of the Reactome pathway [18]. For achieving a normalized enrichment score, gene set permutations were performed 1000 times. *p*-value was set as a significance threshold. Moreover, ssGSEA was conducted using the R package “GSVA”, which generated an enrichment score to signify the levels of absolute enrichment of a metagene set within a given dataset in each sample, which was applied to quantify the enrichment degree of transforming growth factor-β (TGF-β) signaling pathways.

### Evaluation of Immune Status and Immune Cells Relativity

The immune score was calculated with the ESTIMATE algorithm using the R package “ESTIMATE” [19]. The immune scores represent the degree of immune cell infiltration. To evaluate the relative abundance of immune infiltrates, we applied the CIBERSORT R package [20]. The normalized gene expression matrixes of 30 samples were transformed into the composition of infiltrating immune cells. The parameter perm = 1000 was set to estimate the signatures for each sample. Results with *p*-value < 0.05 were considered credible. Then, a bar graph was drawn with the R package “ggplot2” to visualize the content of different types of infiltrating immune cells. Spearman correlation was used to explore the correlation between the stage-specifically expressed genes and the infiltration levels of immune cells. The correlation heatmap was drawn with the “corrplot” package to visualize the correlation between genes and immune cell subtypes.

### Statistical Analysis

All statistical tests were implemented utilizing R software 4.1.0 or Graphpad Prism 8.0. Shapiro–Wilk test was applied to assess data normality and the functions of Shapiro. Test () from the “stats” R package was performed for the normality test. Wilcoxon or Student’s *t*-test was utilized for analyzing the difference between the two groups. ANOVA was utilized for analyzing the difference among multiple groups comparison. All statistical *p*-values were two-sided, and *p*-value < 0.05 was regarded as statistical significance. * represents *p*-value < 0.05.

## Results

### Evaluation of Neural Excitability Levels in Epilepsy

**Fig. 2:**
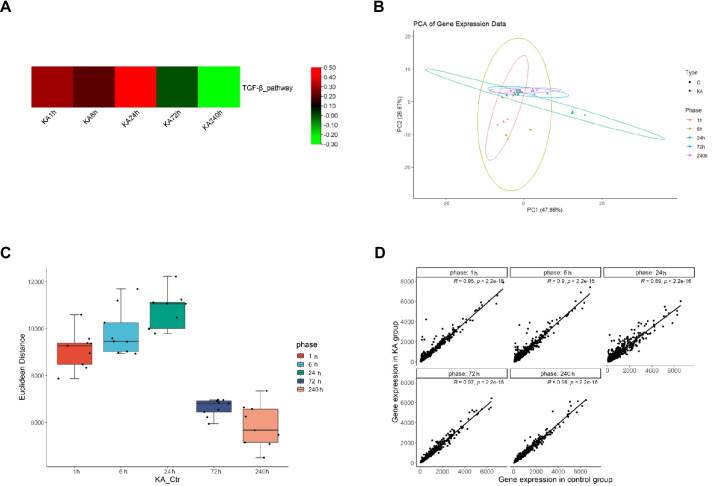
Identification of epilepsy development stage. (**A**) Heatmap shows the enrichment scores of TGF-β pathway in kainic acid (KA)-induced epilepsy brain samples. (**B**) Principal component analysis (PCA) plot shows the transcriptomic data of control and KA-induced epilepsy brain samples. (**C**) Euclidean distance shows the transcriptomic data of control and KA-induced epilepsy brain samples. (**D**) Scatter plots show the correlations between control and KA-induced epilepsy brain samples. C, control; KA, kainic acid

Activation of transforming growth factor-β (TGF-β) signaling is consistent with trends of neural excitability in epilepsy [21]. The transcription levels of genes in TGF-β signaling pathway are regarded as an indicator to reflect neural excitability in epilepsy [22]. To assess the level of neural excitability in the process of epilepsy, we performed ssGESA using the dataset (GSE1834) of kainic acid (KA)-induced epileptic rat model at five time points (1, 6, 24, 72, and 240 h) by calculating the enrichment scores for TGF-β pathway (Fig. 2A). TGF-β pathway genes were determined by the term “transforming growth factor beta receptor signaling pathway (GO:0007179)” in the Gene Ontology database. After species selection, 99 genes were included in the final analysis (Table S1). Gene set enrichment plot demonstrated activation of TGF-β signaling in all time points of epilepsy. The enrichment scores gradually increased and peaked at 24 h, indicating that the highest neural excitability was at 24 h. From 72 to 240 h, the enrichment scores declined, suggesting that the epileptic state tends to relieve. We defined those two trends as the “progression stage” and “remission stage” of epilepsy.

### Assessment of Heterogeneity in Transcriptomic Profiles

Since epilepsy is a time-course process, a multi-level assessment of transcriptomic data was conducted to illustrate the characteristics of overall expression profiles across different stages. The principal component analysis (PCA) of transcriptomic data revealed a clear separation between the control and KA group (Fig. 2B), with the differences gradually increasing within the 1 to 24 h interval. The gene expression profiles of the control and KA group present more homogeneity in the 72 and 240 h interval. We also applied Euclidean distance, an unsupervised clustering method, to quantify gene expression divergence between the control and KA group at different time points (Fig. 2C). The greatest divergence was observed at 24 h, with an increasing trend from 1 h and a decreasing trend after 72 h. The Pearson’s correlation analysis showed similar results (Fig. 2D). The transcriptomes between the control and KA group were more similar from the 72 to 240 h interval than from the 1 to 24 h interval. These results displayed two-part gene expression signatures, which were consistent with the two previously defined stages of the epilepsy process, the progression and remission stages.

### Identification of Genes Specifically Expressed in Different Stages of Epilepsy

**Fig. 3:**
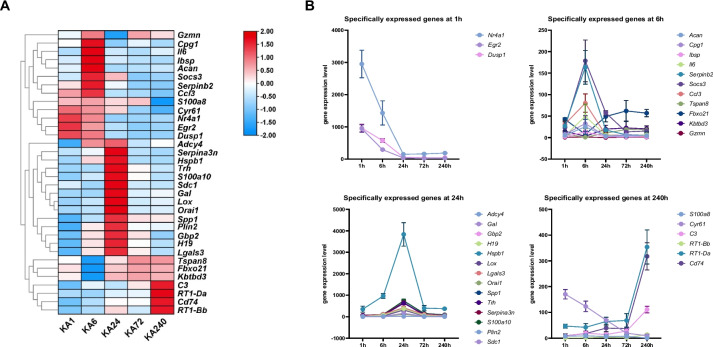
Stage-specifically expressed genes in a time-course progression of epilepsy. (**A**) A clustered heatmap representing the stage-specific gene expression levels. Blue stripes indicate a low expression level while the red stripes indicate a high expression level. (**B**) Trends of the expression level of specifically expressed genes at different time points

Typically, specifically expressed genes can reflect the unique biological state of disease development. As the epilepsy process can be divided into two relatively independent stages, we explored the molecular features of the progression and remission stages by analyzing stage-specifically expressed genes. We used the Tau index, a quantitative, graded scalar measure of the specificity of an expression profile [12, 23], to find genes specifically expressed in certain time points. The Tau index of the genes in all stages was calculated (Table S2), and 34 genes with a Tau index value ≥ 0.8 were selected for the following analysis. As shown in the heatmap of gene expression values (Fig. 3A), different genes were highly expressed at a certain time point (except at 72 h) yet expressed at a relatively low level at other time points. The expression tendencies of genes with the highest or lowest expression at 1, 6, 24, and 240 h were shown in Fig. 3B. The number of specifically expressed genes at the progression stage (1, 6, and 24 h) was greater than that at the remission stage (72 h and 240 h), indicating that more complex molecular mechanisms were involved in the progression stage. The STRING analysis and CytoHubba were used to explore the interaction network of stage-specifically expressed genes, and revealed the hub modules of the PPI network **(**Fig. S1**)**. The top 5 hub genes included interleukin 6 (*Il-6*), secreted phosphoprotein 1 (*Spp1*), aggrecan (*Acan*), dual specificity phosphatase 1 (*Dusp1*), and Galectin 3 (*Lgals3*).

### Validation Datasets of Genes in the Progression and Remission Stages

**Fig. 4:**
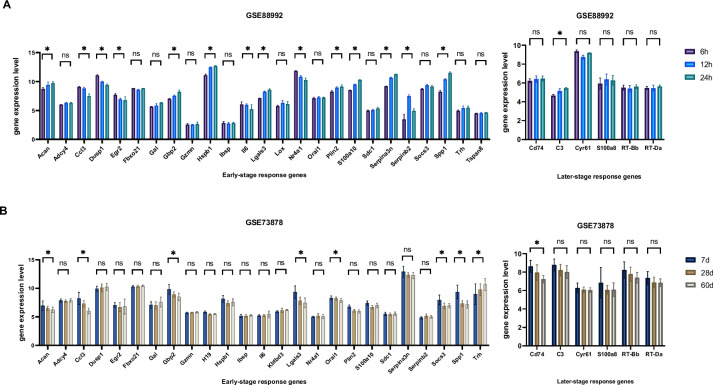
(**A**)Expression Changes of stage-specifically expressed genes in GSE88992 Dataset. (**B**)Expression Changes of stage-specifically expressed genes in GSE73878 Dataset

To validate that the expression of the selected 34 genes have changed in different stages of epilepsy, we employed two datasets for further analysis. Dataset GSE88992 matches the progression stage including 3 earlier time points (6 h, 12 h, and 24 h after seizure induction by KA), while dataset GSE73878 matches the remission stage including 3 later time points (7 d, 28 d, and 60 d after seizure induction by KA). We examined whether the genes identified with specific expression in the progression and remission stages had similar expression patterns in other KA-induced epilepsy datasets. The data indicates that more than half of the progression stage genes (14 out of 25) presented significant changes in the GSE88992 dataset, while the remission stage genes stayed at stable levels in the same dataset (Fig. 4A). In the GSE73878 dataset, however, more than two-thirds of the progression stage genes (17 out of 25) presented no significant changes (Fig. 4B). The remission stage genes also remained stable. These results suggested that stage-specifically expressed genes indeed varied at the progression and remission stage of epilepsy.

### Profiles of Gene Enrichment Analysis

**Fig. 5:**
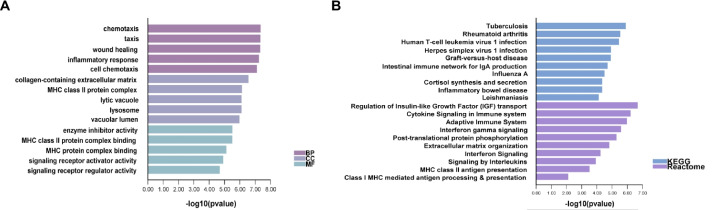
Molecular characteristics of the specifically expressed genes. (**A**) Gene ontology (GO) analysis on biological process (BP), cellular component (CC), and molecular function (MF). (**B**) Pathway analysis on Kyoto Encyclopedia of Genes and Genomes (KEGG) and Reactome

**Fig. 6:**
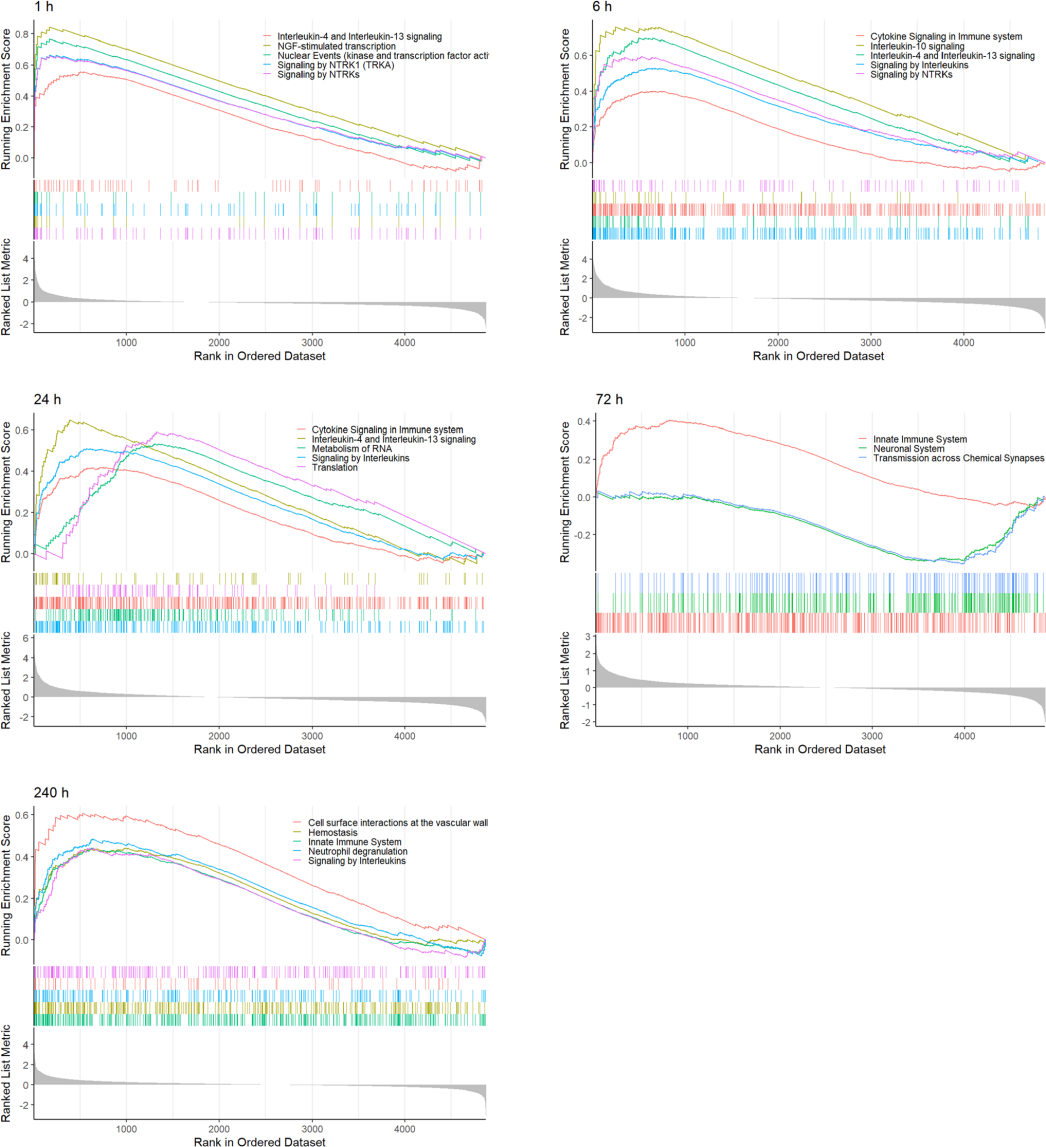
Identification of immune signaling pathways by GSEA at different time points of epilepsy

To explore how stage-specifically expressed genes drive the epilepsy process, we investigated the biological functions and pathways by performing enrichment analyses with GO, KEGG, and Reactome (Fig. 5**, **Table S3) on the selected 34 genes. These genes were shown to be enriched for immune-related functions, including the terms: regulation of insulin-like growth factor-1 (IGF-1) transport, cytokine signaling in immune system, and adaptive immune system. These findings suggested that immune mechanisms may play key roles in the process of epilepsy. Therefore, we further investigated the immune-associated pathways at different time points by GSEA (Fig. 6) and found that different stages of epilepsy were mediated by distinct immune signaling pathways.

### Evaluating the Level of Immune Infiltration

**Fig. 7:**
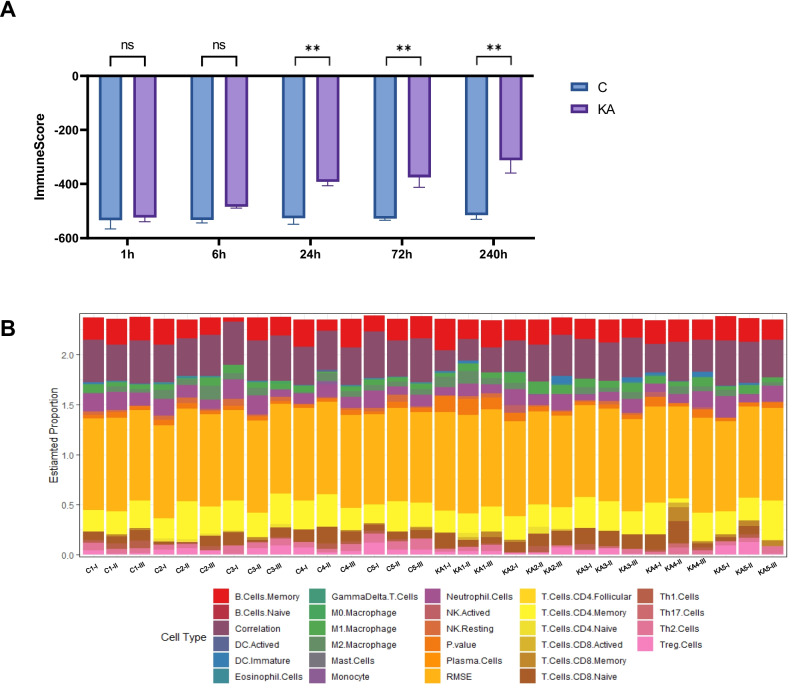
The landscape of immune infiltration in control and epilepsy groups. (**A**) Immune score at different time points. C, control; KA, kainic acid. (**B**) Heatmap of the estimated proportions of each immune cell type

As the stage-specifically expressed genes were found to be highly enriched in immune-related pathways, we next investigated the levels of immune infiltration of samples at different stages. First, we compared the immune score between the control (C) and epilepsy (KA) group. The samples showed similar results at 1 h (*p* > 0.05) and 6 h (*p* > 0.05), while otherwise manifesting significant differences at 24 h (*p* < 0.05), 72 h (*p* < 0.05), and 240 h (*p* < 0.05) (Fig. 7A), which suggested that the immune response may begin to affect the epilepsy process after 6 h. The proportions of immune cell types in the control and KA groups at different stages were furthermore evaluated by ImmuCellAI algorithms (Fig. 7B).

### Immune-Related Gene Regulation with Immune Cell Types

**Fig. 8:**
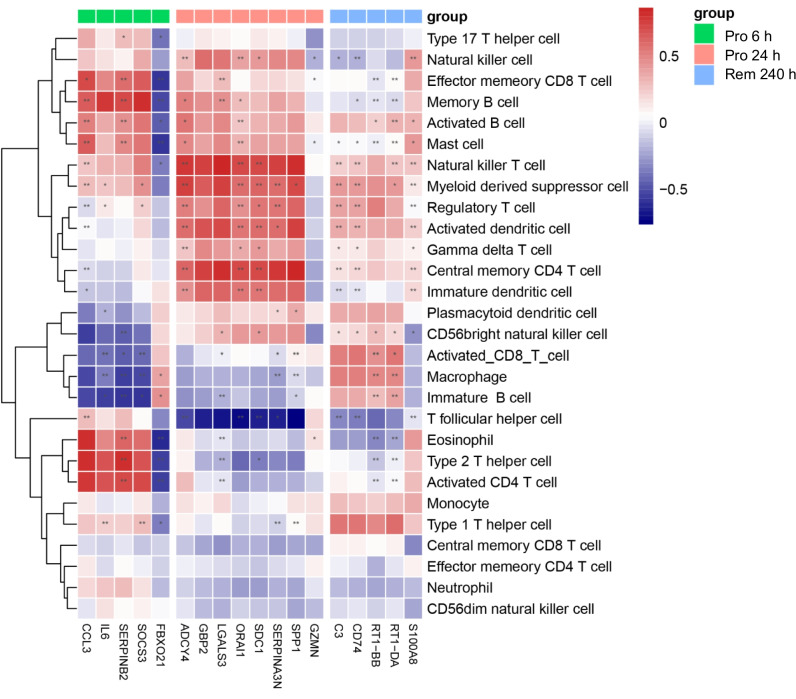
Correlation between stage-specifically expressed genes and infiltrating immune cells. Pro 6 h, progression stage 6 h; Pro 24 h, progression stage 24 h; Rem 240 h, remission stage 240 h

To further explore the roles of stage-specifically expressed genes in immune infiltration, we selected 18 immune-related genes based on pathway enrichment results and literature research (Fig. 8**)**. Interestingly, all immune-related genes were distributed at 6 h, 24 h, and 240 h time points. We then investigated the specific immune cell types that these immune-related genes were involved in during the epilepsy process. The immune-related genes were clustered into 3 subgroups, which matched the time points when they were expressed (6 h, 24 h of the progression stage, and 240 h of the remission stage). Genes of the progression and remission stages activated distinct immune cell types. The genes specifically expressed at 6 h were significantly correlated with activated CD4 T cell, type 2 T helper cell, and effector memory CD8 T cell. At 24 h, genes were significantly correlated with natural killer T cell, central memory CD4 T cell and activated dendritic cell. Genes in the remission stage at 240 h showed relatively low correlation to any cell types. These results indicated that genes from different stages may correlate to different types of immune cells to regulate the process of epilepsy.

## Discussion

Epilepsy is one of the most common neurological diseases, characterized by abnormal excessive or synchronized neuronal activity in the brain [24]. The enduring predisposition of seizures can cause a series of neurobiological, cognitive, psychological, and social consequences [25]. After a seizure, new molecular connectivity mechanisms are composed and form a new order in the brain [26]. Since epilepsy development is a gradual process, analyzing the expression profile characteristics from a time scale perspective can help us reveal more potential information about the progression and remission of epilepsy.

To study the progress of epilepsy, we applied time-course kainic acid (KA)–induced models. The dataset included 1 h, 6 h, and 24 h time points. Since the status epilepticus is completed at 24 h post-KA, the 1 h to 24 h time interval can be defined as the early phase of the disease process [27]. Naturally, the 72 to 240 h time interval can be defined as the chronic phase of epilepsy, due to characteristic electrophysiological features that remain stable in this period [28]. In this study, we first assessed the level of neural excitability with the transcriptomic dataset of KA-induced samples at different time points, which also characterized the severity of epilepsy. We found that neural excitability showed two trends during the epilepsy process, with 24 h after KA induction as an important turning point; it gradually increased until 24 h, after which it declined. To investigate how the molecular mechanisms operate on the time scale relevant to the epoch of increased/decreased activity, we next assessed the overall characteristics of the different temporal expression profiles. We found significant heterogeneity in the KA-induced epileptic samples between the 1 to 24 h interval, and the 72 to 240 h interval, which are defined as two stages: the progression stage and the remission stage. Combining the overall transcriptomic differences and epileptic status, we assumed that the dynamics of gene expression and physiological functions were modulated in a stage-specific manner: epileptic status was exacerbated during the progression stage and relieved during the remission stage.

As the process of epileptic status presents stage-specific features, we aim to discover which genes are involved in forming these features at different stages of epilepsy. These specific genes, referring to those only expressed in unique tissues or conditions, provide important clues about specific biological functions [29]. These genes may serve as potential biomarkers for disease recognition and help us understand the disease’s mechanisms [30]. Therefore, the Tau index of gene expression was calculated to identify specifically expressed genes at the progression and remission stages. As predicted, the expression of several genes previously proved to be associated with epilepsy was indeed stage-specific. Small inducible cytokine A3 (*Ccl3*) [31] and suppressor of cytokine signaling (*Socs3*) [32] were identified to be highly expressed at the progression stage (6 h after KA induction). Complement proteins C3 can tag inappropriate synaptic connections between neurons to elimination by phagocytic microglia during the synaptic pruning period, which enhances the disorder of microglia-astrocyte communication in epilepsy [33, 34]. These findings were also verified in two independent datasets of KA-induced epilepsy models. The genes identified to be specifically expressed at the progression stage were more likely to have a significantly different expression level in early stage (1 h, 6 h, 12 h) datasets (GSE88992) than in late stage (7 d, 28 d, 60 d) datasets (GSE73878). This suggests that these genes can be potential candidates for the prediction of epilepsy progression. However, the expression level of the genes specifically expressed at the remission stage did not differ significantly between the early and late stage datasets, which may result from the long duration of the study and potential distinct mechanisms [35].

By further exploring the biological functions of stage-specifically expressed genes, we noticed that many immune-related pathways were enriched. For example, the major histocompatibility complex (MHC) class II molecules were enriched in both function and pathway terms. In the acute phase of epilepsy, microglia become activated, and the mRNA and protein expression levels of MHC II are increased. In the later phase, the MHC II returns and maintains normal levels [36, 37], indicating that MHC II plays a more important role in the progression stage of epilepsy. Several neuroinflammatory pathways, which are known to contribute to the development and progression of epilepsy, were also enriched. The regulation of insulin-like growth factor-1 (IGF-1) transport was notably one of the most enriched terms. Previous studies have found that IGF-1 levels and IGF-1 receptor activation were increased in human epileptogenic tissues. Increased IGF-1 levels promoted seizure activity via IGF-1R-dependent mechanisms and Akt-mTOR signaling [38, 39].

Moreover, the rapid release of proinflammatory cytokines and activation of immune signals are observed after acute seizures in both experimental and clinical settings. On the other hand, cytokines and other inflammatory mediators are chronically overproduced during chronic epilepsy, implying the neuromodulator role of the immune system and its potential involvement in the generation of spontaneous seizures [40]. We additionally noted the enrichment of several types of cytokines in our findings, such as interferon-gamma (IFN-γ) and interleukins (IL). Previous studies have shown that IFN-γ was elevated in the serum and cerebrospinal fluid (CSF) in epilepsy patients [41]. *IL-6*, the hub gene in PPI analysis (Fig. S1), is significantly increased in the epileptogenic brain tissues [42–44]. As a neurotoxic cytokine, IL-6 is mainly produced by microglia and astrocytes, then choosing to bind its receptors onto neurons in brain regions such as the thalamus, hippocampus, and cortex [45]. Upon pathological changes like epilepsy occurring, IL-6 secretion capacity will be enhanced by the activated microglia [46]. IL-6 showed high expression at the 6 h interval (Fig. 3A), indicating a strong immune response of microglia and astrocytes at this time point.

As the specifically expressed genes are primarily enriched for immune functions, we speculated that immune mechanisms might play a key role in the pathogenesis of epilepsy. Both innate and adaptive immune responses are promptly induced within the CNS to deal with pathogenic stimuli, self-antigens, and various etiologies [47]. These immune responses are not only present in epilepsies caused by infectious and inflammatory diseases, but also in epilepsies without clear inflammatory pathophysiology [48–50].

Recent reports have shown that a relatively acute innate immune response appears within 1 to 3 days, yet later disappears after 7 to 14 days [51]. However, in our results, we found that the earliest immune response appears less than 24 h after the induction of epilepsy. Neural activity and the variability of expression profiles in the epilepsy group were reduced during the “remission stage”, which was from 72 to 240 h. However, the level of immune infiltration was gradually increased during this period compared with the control group, suggesting that recovery to normal brain function is associated with enhancing the immune response, although it might happen through other immune mechanisms distinct from those in the progression stage, which has been confirmed by GSEA analysis (Fig. 6).

Differences in immune response mechanisms during different stages of epilepsy may also result from the recruitment of different immune cells. We then explored which immune cell types were regulated by the stage-specifically expressed genes in different stages of epilepsy. The genes at the same time point manifested a similar correlation with the immune cell types. From the 1 to 6 h time points, genes were highly correlated to CD4 T cells and eosinophils, while the degree of immune infiltration remains at relatively low levels **(**Fig. 7A**)**. This may be due to the limited recruitment of immune cell types at an early stage. However, in 24 h samples, stage-specifically expressed genes showed a significant correlation with natural killer cells, which is consistent with the findings in epileptic patients [52]. These results indicate that both innate and adaptive immunity were involved in the progression stage of epilepsy, although the immune cell types were different. In the remission stage, genes presented a relatively low correlation with any cell types, implying more complicated mechanisms might be involved in the immune response during this stage of epilepsy.

Epilepsy stage development is a gradual process with multiple dynamically changed molecular mechanisms involved. By studying the molecular features of stage-specific expression profiles, we can understand the disease mechanisms and identify the key factors involved in the epileptic process, which may shorten the chronic course of epilepsy when treated as therapeutic targets. Immune regulation is a complex process in disease. Early immune activation may help the brain to cope with different factors of injury, while later immune activation may be a key mechanism to enhance brain repair. From our study, these results indicated an essential role of immune regulation as the potential mechanism of epilepsy development. Moreover, epileptic status in different periods may be mediated by different immune mechanisms resulting from specifically expressed genes regulating specific cell types. The findings of our current study are compelling, but they have some limitations. The lack of exact-match time points in validation datasets may limit the interpretation of the results, especially in the remission stage. Although our findings highlight the relationship between key genes and the epilepsy progress, we should not ignore that the immune mechanism may be a double-edged sword in disease progression, and more evidence is necessary to verify whether key genes are beneficial or detrimental to the disease in specific stages.

### Supplementary Information

Below is the link to the electronic supplementary material.

Supplementary file1 (PDF 136 KB)

Supplementary file2 (XLSX 10 KB)

Supplementary file3 (CSV 147 KB)

Supplementary file4 (XLSX 13 KB)

## Data Availability

The data sets supporting the conclusions of this article are available in the public databases the Gene Expression Omnibus (GEO, https://www.ncbi.nlm.nih.gov/geo/) with the accession numbers: GSE1834, GSE88992, and GSE73878. All these studies have been previously approved by their respective institutional review boards.
