# Supplementary figures and images for: Transcriptional Signatures of a Dynamic Epilepsy Process Reveal Potential Immune Regulation
Source: Mol Neurobiol. 2023 Nov 22;61(6):3384–96. doi: 10.1007/s12035-023-03786-x (PMC11087345; doi:10.1007/s12035-023-03786-x)

**A**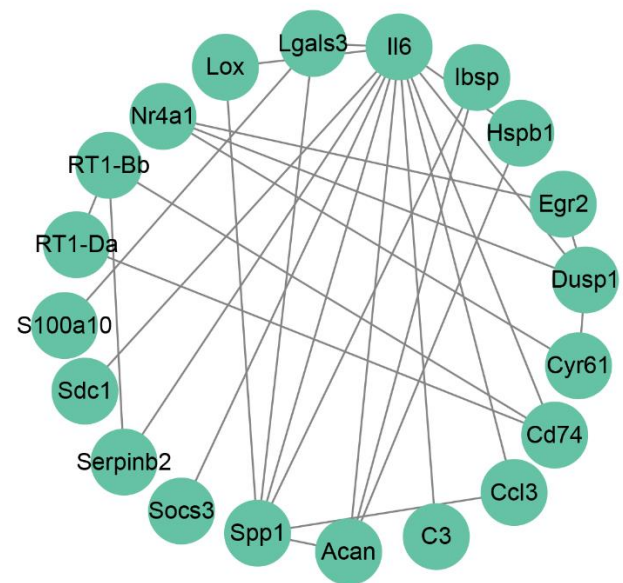**B**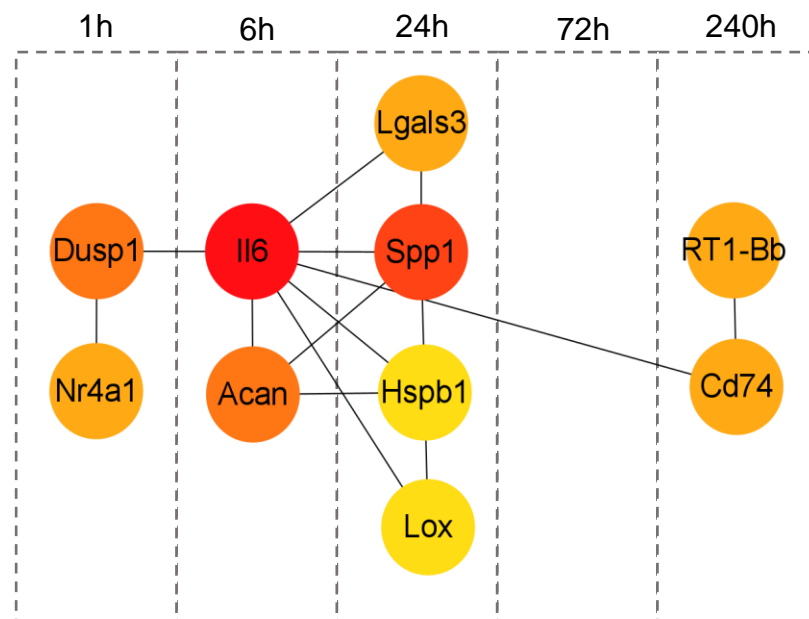

Supplement: Supplementary file 1 — Supplementary file1 (PDF 136 KB) [file 12035_2023_3786_MOESM1_ESM.pdf]
